# An approach to semantic query expansion system based on Hepatitis ontology

**DOI:** 10.1186/s40709-016-0044-9

**Published:** 2016-07-04

**Authors:** Chen Yunzhi, Lu Huijuan, Linda Shapiro, Ravensara S. Travillian, Li Lanjuan

**Affiliations:** Zhejiang University School of Medicine, Hangzhou, China; Hangzhou Vocational and Technical College, Hangzhou, China; College of Information Engineering of China Jiliang University, Hangzhou, China; Computer Science and Engineering, University of Washington, Seattle, WA USA; Zhejiang University the First Affiliated Hospital, Hangzhou, China

## Abstract

**Background:**

Ontology development, as an increasingly practical vehicle applied in various fields, plays a significant role in knowledge management. This paper, focusing on constructing and querying a hepatitis ontology, aims to provide a framework for ontology-based medical services. The paper is devoted to the algorithm of query expansion for the hepatitis ontology, including synonym expansion, hypernym/hyponym expansion and expansion of similar words. It applies semantic similarity calculation to judge the similarity of retrieval terms.

**Results:**

The paper proposes a new prototype system. The accuracy of query expansion is improved in both precision@40 and AP@40, which indicates that query expansion improves the accuracy of the query after using the method proposed in this paper.

**Conclusions:**

The paper has adopted semantic similarity computing to improve retrieval performance. Experiments show that search precision of query expansion is higher based on domain concept relationship.

## Background

Derived from the field of philosophy, an ontology is a explanatory or descriptive model of a system, which represents entities and relationships among entities in that model [1]. It refers to the semantic basis for the communication between subjects (human, machine, software, etc.) within a domain. In other words, an ontology offers a basis for shared knowledge. An ontology primarily provides services for machines which do not understand the semantics of the natural human language, because the current computer can only deal with the text as a string. Therefore, an ontology, as a machine-understandable, formally specified and shared representation of domain knowledge provides a description of meta-knowledge and is considered to be a powerful tool in knowledge management. Ontologies represent the common recognition of the vocabulary in a certain domain and give a clear definition of terms and the relationships between terms at different levels of a formal model—for example, the establishment of terms and the relationships among terms in medical disciplines.

Medical ontologies have been developed to support a number of areas in medicine [2]. Examples include high level ontologies such as the unified medical language source (UMLS), and the systemic nomenclature of medicine (SNOMED.), etc.

Ontologies and terminological resources have appeared in information retrieval (IR) to provide query expansion terms [3]. Query expansion refers to the addition of more related terms and phrases to the original query terms in retrieval, by applying computer linguistics, informatics, and other technology, to generate new and more accurate query terms for the user. These expanded query terms, used in the re-retrieval, are expected to improve the accuracy of information retrieval, resolve term-mismatch problems and provide the user with sufficient information [4].

Hepatitis is an infectious disease seriously hazardous to human health and China is a highly epidemic area of viral hepatitis [5]. This paper documents the establishment of a liver disease ontology in order to conduct research on query expansion.

This paper is organized as follows: “Construction of the hepatitis ontology” section “Semantic retrieval from the hepatitis ontology” section, “Results and discussion” section and "Conclusions".

## Methods

### Construction of the hepatitis ontology

#### Ontology construction principles

The constructed ontology should follow the principles of clarity, consistency, integrity, and scalability [6]. Clarity means that the ontology terms should be defined unambiguously; consistency refers to that the relations between the terms should be logically coherent; integrity refers to that the concepts and relations should be complete and include all concepts in the domain; and scalability requires that the ontology should be extendable and open to a new concept with the continuous development in this domain.

In the field of medical knowledge engineering, the organization and management of medical knowledge is of great importance. Traditional methods for organizing and managing medical knowledge mainly rely on applying the rigorous and standardized classification and MeSH (medical subject headings) concurrently [7]. However, nowadays, the ontology based on natural language is considered preferable. As medical science is a disciplinary science and the terminologies in this field are specialized and comprehensive, purely natural language cannot usually be adopted in ontologies. Instead, the standardized language in MeSH and newly emerging natural languages which have not yet been enrolled in MeSH, were adopted to construct the vocabulary of the hepatitis ontology. Relying on the preference relationships in MeSH, the matching relationships, the relationships between the entry terms and headings, as well as headings and sub-headings, we aim to build a network of keywords in the medical domain by taking advantage of ontology.

#### Hepatitis ontology design

This research, with special reference to hepatitis, a class of infectious diseases, aims to extract some core concepts and instances as well as relevant relationships, so as to construct an experimental ontology knowledge base. This knowledge base, in turn, helps us to make use of the features of medical ontologies, explore the principles of medical research and elucidate the method of constructing ontology and its retrieval characteristics. The project is expected to provide references for medical ontology-based services and offer intelligence support for medical experts’ working systems, information retrieval, and medical education, as well as comprehension of natural languages.

At present, there are many existing ontology knowledge bases. The process of ontology construction varies with their respective fields and specific engineering considerations. This research does not simply choose an ontology construction method as a way to guide the development of hepatitis ontology, but rather, specifically applies the “seven-step method” [8].

### Workflow

**Fig. 1:**
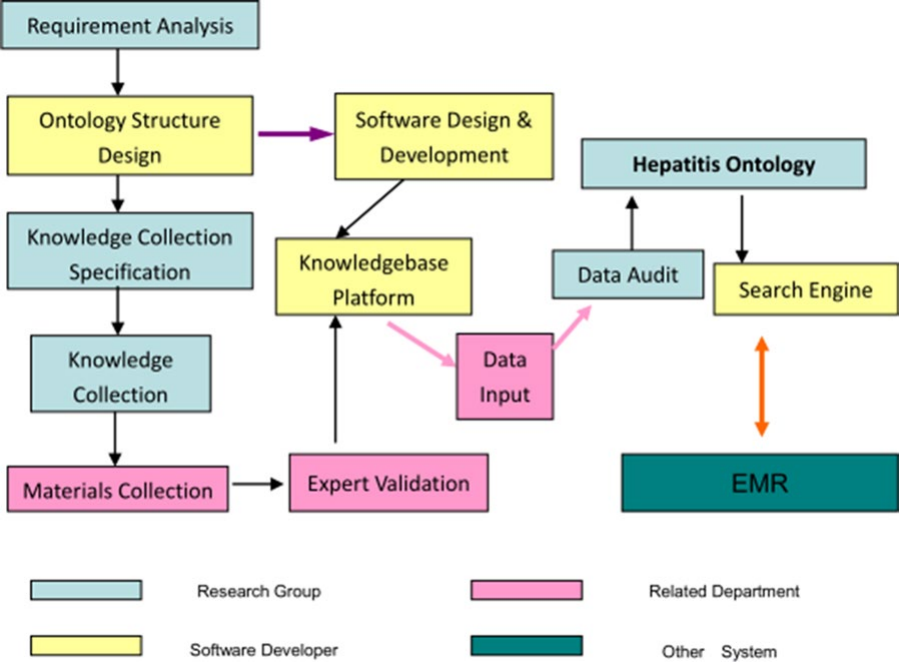
Workflow of hepatitis knowledgebase

The workflow of constructing the hepatitis ontology knowledge base is shown as Fig. 1.

The aim of the requirement analysis phase is to determine the main functions of the knowledge base. The structure design phase concerns how to design the ontology of hepatitis diseases, which is the focus of this research. In the phase of knowledge collection specification, the main task is to formulate standards about how to collect knowledge and where to find knowledge, writing format, content, reference resources, etc.

After the training of knowledge collection specification, researchers begin to collect all kinds of materials. Afterwards, in the phase of expert validation, relevant department experts evaluate whether the gathered materials meet the requirements for the content of the knowledge base. According to the results of the structure design phase, software developers design and build a knowledge base platform. After having finished data input and data audit, researchers have a completed knowledge base. In order to facilitate other applications (e.g., EMR), the researchers still needed to design a search engine for external applications. In the whole work process, different researchers were respectively responsible for different tasks and responsibilities, jointly completing the design work of a knowledge base.

### Concept analysis of hepatitis

To construct the domain ontology, researchers should first determine the key concepts, such as the upper-level concept and the commonly used concepts in this field, in order to establish the core concept sets. Using hepatitis, for example, to construct an ontology model, the researchers focused on the definition of the core concept and the key concepts, and on describing the relations between the core concept and other concepts.

Concept extraction mainly refers to the merging, classification, and semantic analysis of the vocabulary and terms in the literature. By merging, we mainly refer to putting synonyms together as different terms describing the same concept. For instance, terms such as HB, Hepatitis B, serum hepatitis, etc. actually refer to the same disease. Classification means merging the terms into its upper-level concept, making it a sub-concept or an instance of the upper-level concept according to the medical and health volume of CLC (Chinese library classification) and the classification table of MeSH. For example, hepatitis C is a sub-concept of hepatitis and an instance of it as well. Semantic analysis includes three aspects:

1. Analyze the part of speech of the term. Nouns and verbs are the two major parts of speech under our consideration.
2. Understand the annotations of the term to identify the exact meaning of the term and its category.
3. Think over the multiple meanings of the term in various contexts and the most appropriate meaning of a term in a certain context. For example, “therapy” has two kinds of part of speech: nouns and verbs, the term “therapy” in Chinese can mean the therapeutic measure and the medicine for a kind of disease, also mean to cure a type of a disease.
  The semantic analysis resulted in concepts and a terminology set.

### Constructing the hepatitis ontology framework

After carrying out the concept analysis of hepatitis, we began to design hepatitis ontology structure. The ontology editor tool used is Protégé 3.4.1, which was developed by Stanford Center for Biomedical Informatics Research of the Stanford University School of Medicine.

In addition, in the field of medicine, the following aspects should be noted concerning ontology construction [9]:

- Abstract classes should not be under concrete classes. According to the principle of class inheritance, it is clearly incorrect if an abstract concept inherits a representative of concrete concepts.
- The whole class should be as evenly distributed as possible. The classes are designed according to object-oriented design principles. If classes are unevenly distributed, it may bring additional overhead to class management and maintenance.
- Class inheriting cannot be cyclic. If class A inherits from class B, class B inherits from class C, and class C inherits from class A, then the class inheriting is cyclic and such a cycle must be avoided.
- Class naming is unique. Each class represents the characteristics of particular entities, so each kind of class name should points to a unique class. But the same kind of thing can have different class names. For example, “hepatitis B” has another name of “serum hepatitis”.
- The range is limited. It should be noted that an ontology is unlikely to include all information in a specific domain. Ontologies are not supposed to contain all possible attributes of all classes and all essential distinctions between classes. It is reasonable to present only the most obvious attributes, rather than all the relationships between terms in an ontology.
- Regarding the instances and categories, it is not always an easy thing to determine in a specific ontology whether a concept is a class or an instance of a class, depending on the precise expression terms in ontology. The smallest single instance is expressed as the most detailed concept in the knowledge base.
- The vocabulary for describing the professional knowledge should be specialized. Characterized by medical knowledge, the vocabulary of ontology should accord with medical language. The credibility of the source of knowledge should be high (preferably national standards or international standards). Knowledge coverage should be wide and outmoded knowledge should not be incorporated.

After the semantic analysis, we obtained the following concepts and terminology set:

1. *Disease* Instances: hepatitis A, hepatitis B, hepatitis C, hepatitis D, hepatitis E.
2. *Background* Causes; pathogenesis; epidemiology: incidence and prevalence, demographics, etc.
3. *Diagnosis* Clinical presentation: symptom, sign; lab examination; differential diagnosis; diagnosis ideas: diagnostic decision, questions to ask, guidelines, consider consult, etc.
4. *Treatment* Immediate action; therapeutic options: summary of therapies, medications and other therapies, efficacy of therapies, evidence-based medicine; coexisting disease; special patient groups; therapies consult; informed consent; follow-up, etc.
5. *Prevention* Primary prevention; secondary prevention, etc.

**Fig. 2:**
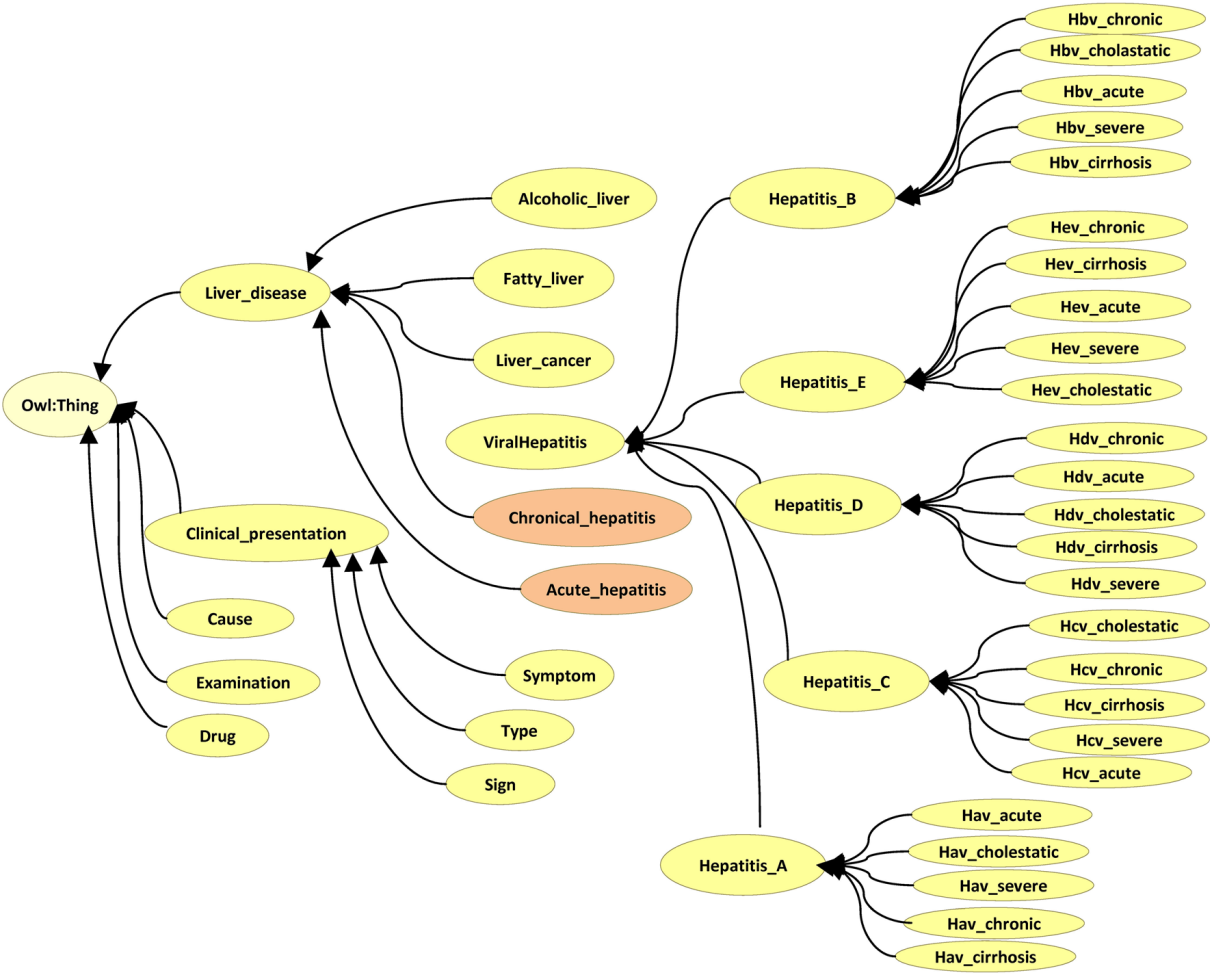
Hepatitis ontology structure (asserted model, drawn by OWLviz)

**Fig. 3:**
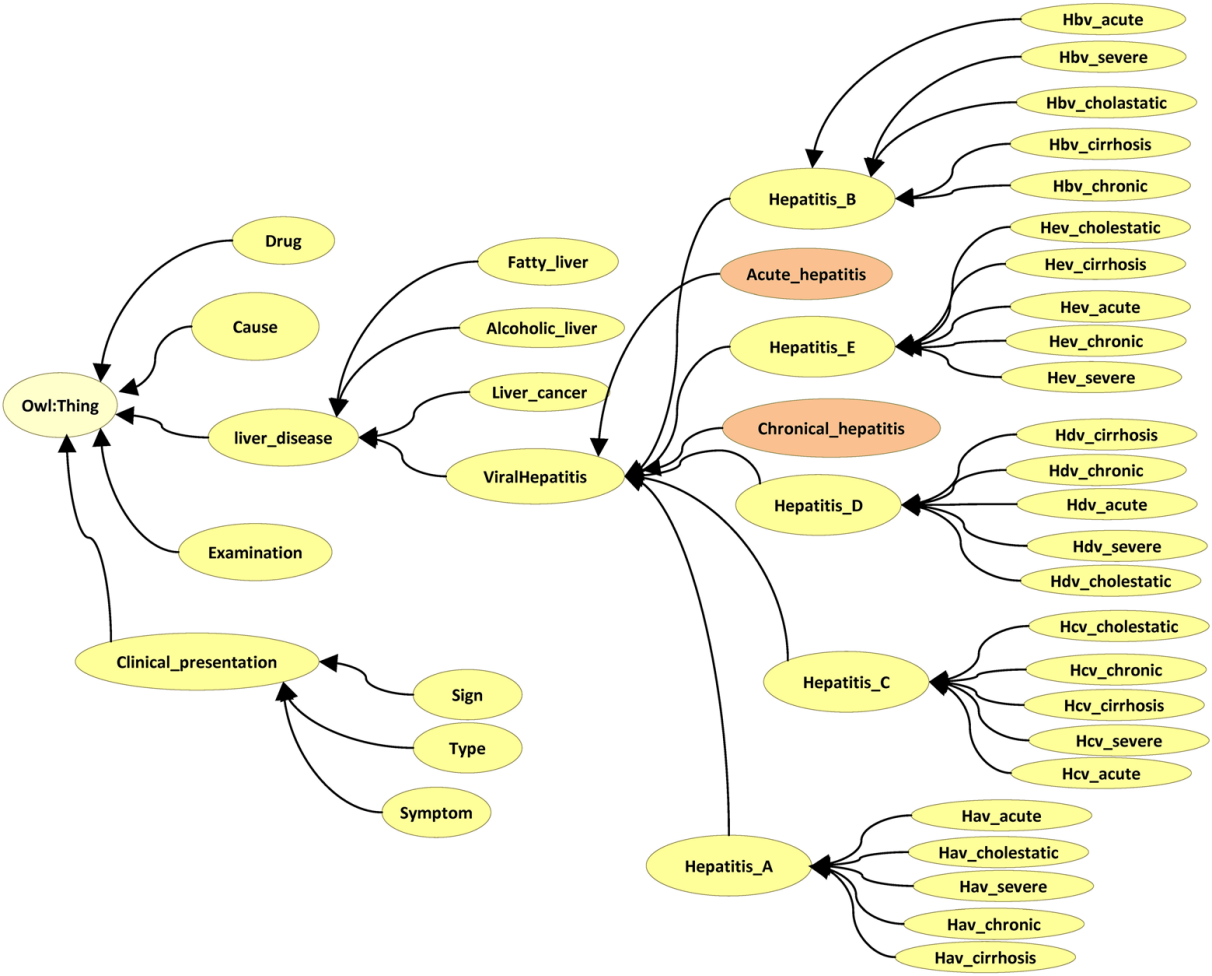
Hepatitis ontology inferred hierarchy

**Fig. 4:**
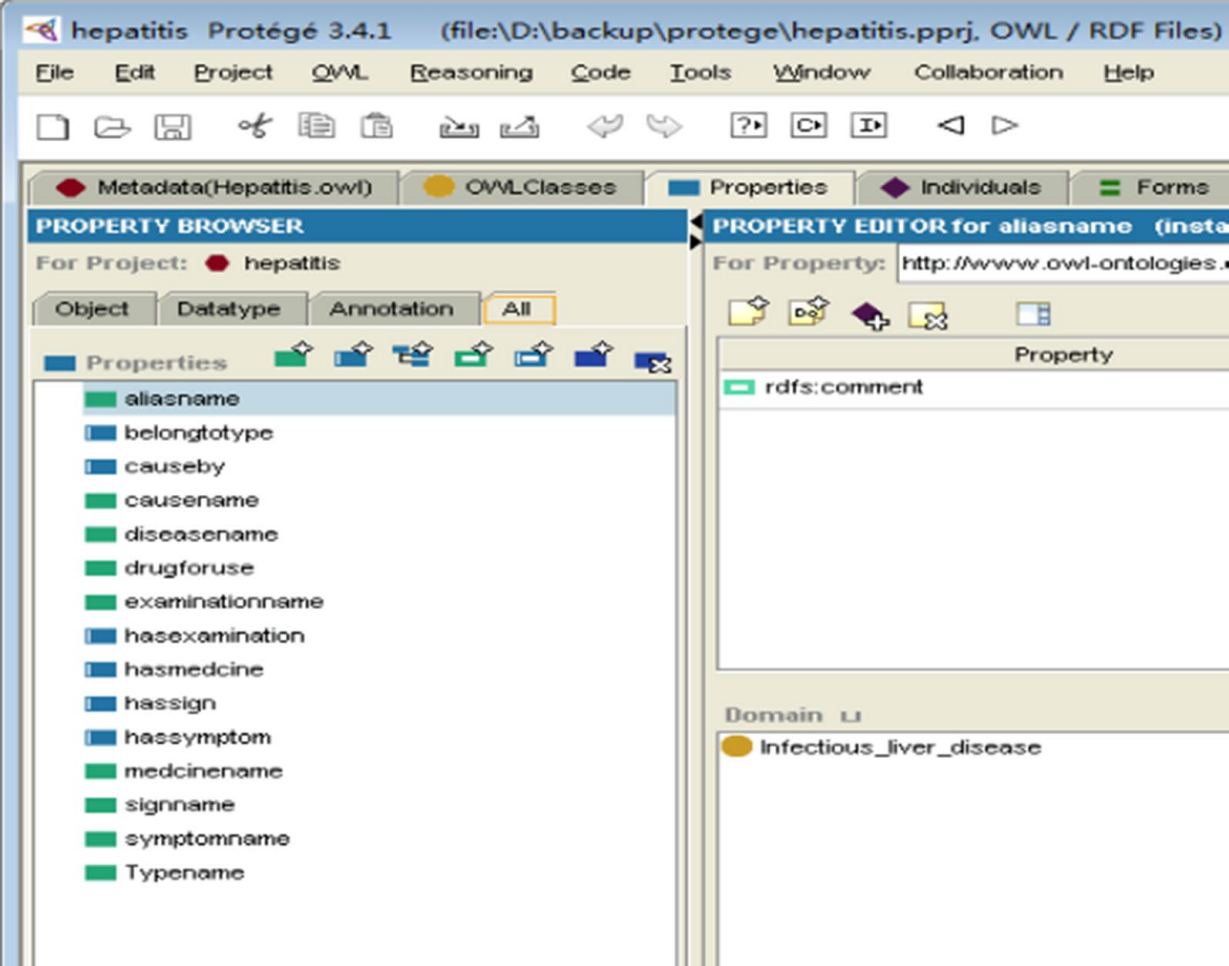
Properties of hepatitis ontology

We used the ontology editor tool Protégé to define classes and class hierarchy. The ontology hierarchy is shown in Figs. 2 and 3. The ontology class hierarchy shows the relationships among upper-level and lower-level classes. The next step was to define the class attributes. In order to facilitate intelligent retrieval based on semantic understanding, close attention should be paid to synonyms in setting class properties. As natural language is very rich, the same things tend to have multiple synonymous terms, including. For example, “infectious liver disease” means “viral hepatitis”. The hepatitis ontology property is shown in Fig. 4.

**Fig. 5:**
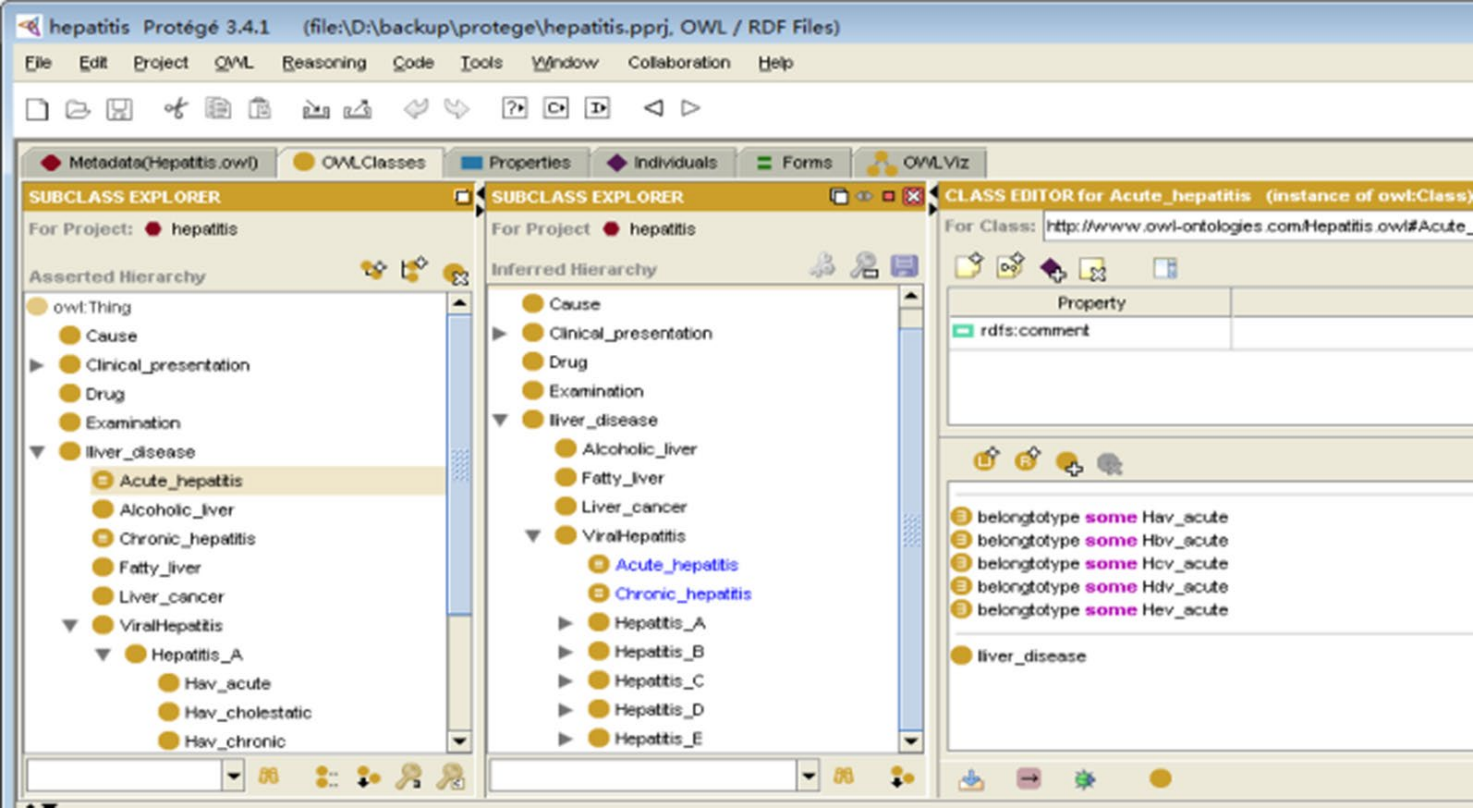
Asserted hierarchy and inferred hierarchy of hepatitis ontology

In Protégé-OWL, classes that have at least one set of necessary and sufficient conditions are known as defined classes. Classes that do not have any sets of necessary and sufficient conditions (they have only necessary conditions) are known as primitive classes. In Protégé-OWL, defined classes have a class icon with an orange background. Primitive classes have a class icon that has a yellow background. In Figs. 2 and 3, the classes “acute_hepatitis” and “chronic_hepatitis” belong to defined classes with an orange background. These two classes are described by five conditions, shown in the right side of Fig. 5. The classes “acute_hepatitis” and “chronic_hepatitis” in the asserted hierarchy (manually constructed hierarchy) are subclasses of “liver_disease”. Classes that have had their superclasses changed by reasoning are shown in blue.

### Semantic retrieval of hepatitis ontology

Traditional retrieval technology is based on keyword matching. The search word that users input is not understood from the semantic level. Query expansion was subsequently introduced to information retrieval [10], enabling more keywords than the initial ones by extending keywords effectively, such as synonyms, near-synonyms and similar words. By sending these expanded terms into a search engine to retrieve again, results more closely meeting users’ requirements can be obtained.

Incorporating relevant research concerning query expansion method, we propose an improved semantic query expansion method based on domain ontology [11].

#### Ontology semantic retrieval process

**Fig. 6:**
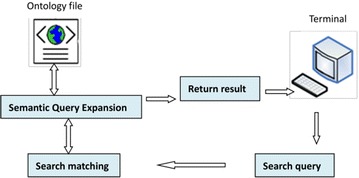
Ontology retrieval process

As mentioned above, the intelligent semantic retrieval system can solve many problems of traditional information retrieval, retrieve the relevant information that users’ searches request, and realize intelligent retrieval functions. The ontology retrieval process consists of users inputting search queries, matching search queries, expanding semantic queries based on the ontology content, and returning relevant results. This process is shown in Fig. 6.

#### Semantic query expansion algorithm

**Fig. 7:**
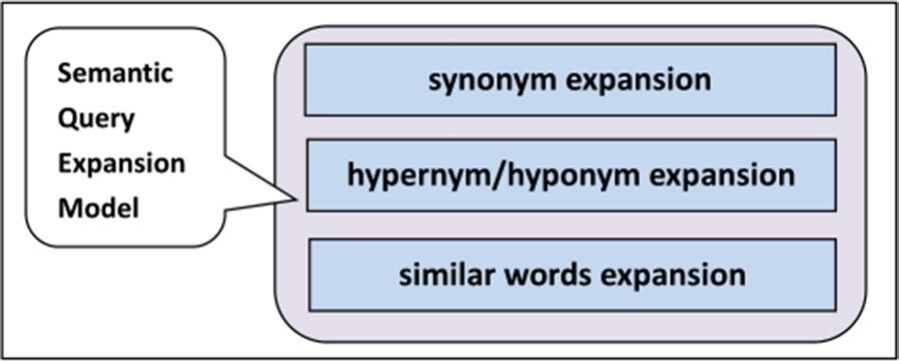
Semantic reasoning model

Common relations in ontology generally include hypernym/hyponym relationships, peer relationships, and synonymy relationships. Based on these relationships, Na [12] and Ming [13] designed a semantic query expansion model, shown in Fig. 7, which we utilized. Semantic reasoning includes synonym expansion, hypernym/hyponym expansion and similar words expansion. Synonymy means that words with the same meaning or similar meaning can often be replaced by each other. For example, “infectious liver disease” is equal to “viral hepatitis” and “hepatitis B” is equal to “serum hepatitis”. Hyponym term is a special instance of hypernym term, just like the relationship between animal and tiger or elephant. Tiger, being a kind of animal, is an instance of animal. Sometimes using hypernym/hyponym in the search can help to retrieve potentially useful information such as the relationship between viral hepatitis and hepatitis A, hepatitis B, hepatitis C, hepatitis D and hepatitis E (see Fig. 2). Similar words have similar meaning. For example, hepatitis C and hepatitis B, these two words are neither synonym nor hypernym/hyponym, but they all are closely related to hepatitis.

**Fig. 8:**
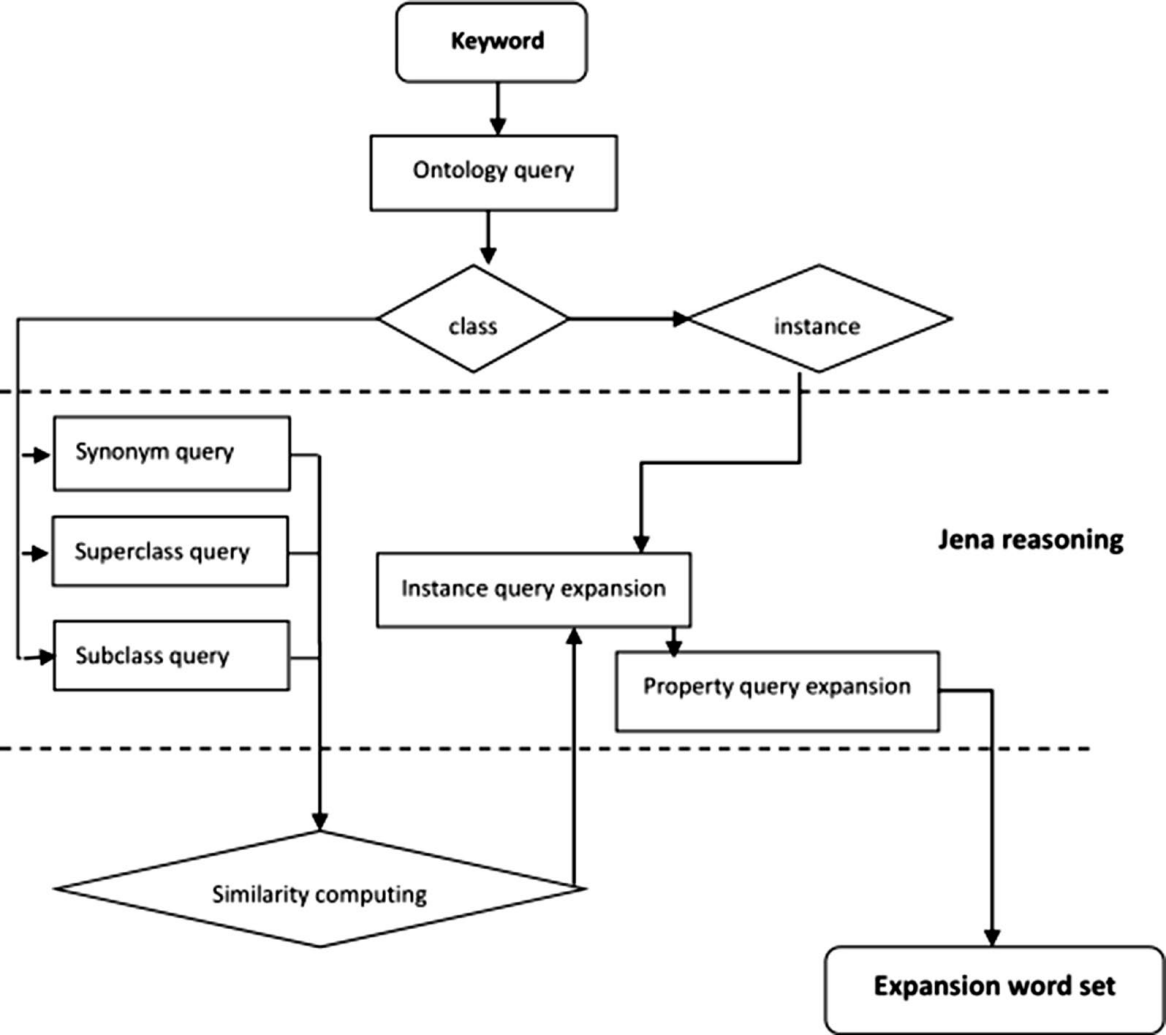
Semantic query expansion process

Based on the model in Fig. 7, we designed the query expansion process shown in Fig. 8.

The retrieval process consists of the following steps.

The user inputs the search keyword, and then the system tests whether the keyword belongs to a class or an instance.

If the keyword is a class name, then the system carries out query expansion that is relative to class, such as synonym expansion and hypernym/hyponym expansion. If the keyword is an instance name, then the system carries out instance query expansion. Meanwhile, the system will look for relative class name or instance name according to the particular properties of the keyword.

After synonym expansion and hypernym/hyponym expansion, the system starts to compute similarity in order to decide whether the results should be returned to the user, according to the similarity threshold value.

The system outputs a word set of the results after query expansion.

Step (2) uses the Jena environment, a semantic web tool, which can read, write and process ontology data created by Protégé. The pseudo-code of the query expansion algorithms process is as follows:

#### Semantic similarity computing

An important step in the process of semantic query expansion (in Fig. 8) is the semantic similarity computing.

At present, scholars have made abundant achievements in the researches on the semantic similarity calculation method based on ontology. Ontology concept structure can be described as a hierarchical tree, in which nodes of the ontology are concept words, and the edge is the relationship between ontology concepts. In general, broad conceptual domains are located in higher positions in the tree structure, and its node density is low. By contrast, specific conceptual domains are located in lower positions in the tree structure, and its node density is relatively high. Therefore, the concept semantic similarity calculation in the tree structure was mainly affected by factors such as node depth, node density, and distance between nodes.

Xin [14] proposed a method of semantic similarity computing based on the tree structure. Considering the features of the tree structure of the hepatitis ontology, this paper proposes a modified similarity computing method on the basis of Xin. The computing method in this paper is much simpler than Xin’s mathematical calculation. The semantic similarity computing formula is shown as follow.

I $$ Sim\;(A,B) = \alpha \times Dist(A,B) + \beta \times Depth(A,B) + \lambda \times Density(A,B) $$

Sim (A, B) stands for the semantic similarity between A and B.

Dist (A, B) expresses the semantic distance between two concept nodes. Semantic distance refers to the edge number of the shortest path between two nodes in the tree structure. Because the semantic distance is inversely proportional to semantic similarity, the calculation method is as follows:

II $$ Dist\;(A,B) = e^{ - dist(A,\;B)} $$

Linguistic research suggests that the greater the semantic distance of two words is, the lower its similarity is. In particular, when the semantic distance between two words is zero, the similarity is 1. When the semantic distance of two words is infinite, the similarity is 0.

Depth (A, B) expresses depth relationship between two nodes in ontology. The expression is as follow.

III $$ Depth(A,B) = \frac{|depth\;(A) - depth\;(B)| + 1}{depth\;(A) + depth\;(B)} $$

Node depth refers to the edge number of the shortest path from the concept node to the root node. Given the same semantic distance, the greater the depth difference of the two nodes is, the smaller the similarity between concepts is. Conversely, the smaller the depth of the two nodes is, the greater the similarity between concepts is.

IV $$ Depth\;(A) = s + 1 $$

In formula (IV), depth (A) expresses the edge number of the shortest path between concept A and the root node. The depth of the root node is 1.

Density (A, B) expresses node density between two concept nodes, which stands for the child node density of the common ancestor node between two nodes. Generally speaking, the greater the density of child node is, the more concretely refined the concept is. The expression of node density is as follows:

V $$ Density\;(A,B) = \frac{n}{m} $$

In formula (V), n stands for the number of direct child nodes of the common ancestor node between A and B. m stands for the number of all child nodes of the common ancestor node between A and B.

**Fig. 9:**
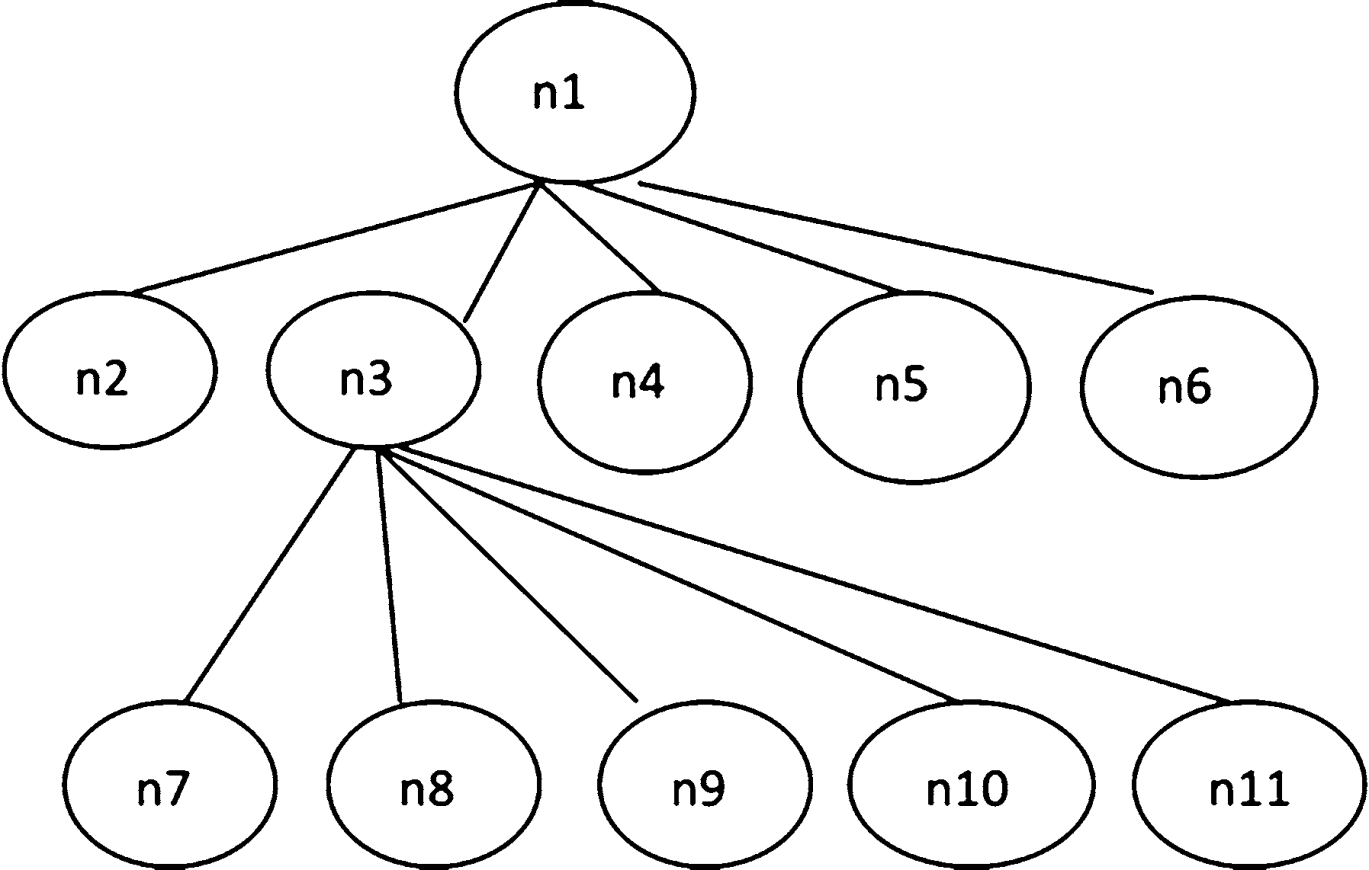
Part structure of hepatitis ontology

Choosing part of the hepatitis ontology in Fig. 2 to analyze semantic similarity, we get a branch of hepatitis as shown in Fig. 9.

In Fig. 9, n1 represents the node “viral hepatitis” in Fig. 2. The detailed mapping between Figs. 2 and 9 is listed in Table 1. According to the ontology structure in Fig. 9, using semantic similarity calculation formula (I) described earlier, we get the similarities between each node as shown in Table 2. The parameter in formula (I) is set as *α* = 0.6, *β* = 0.2, *γ* = 0.2. From Table 2, the similarity between n1 and its child nodes (n2, n3, n4, n5, n6) is 0.4541 while the similarity between n3 and its child nodes (n7, n8, n9, n10, n11) is 0.5007. It is clear that “hepatitis_B” is very similar to its child class i.e. “Hbv_acute”, “Hbv_chronic”, “Hbv_server”, “Hbv_cholestatic” and “Hbv_cirrhosis”. Moreover, compared to n1 and its child nodes (n2, n3, n4, n5, n6), the similarity between n3 and its child nodes (n7, n8, n9, n10, n11) is greater. That is to say, the farther the child nodes are from their root node, the more similar the child nodes are to their parent nodes.

**Table 1:** Node name mapping Table of Figs. 2 and 9

Fig. 9	Fig. 2
n1	ViralHepatitis
n2	Hepatitis_A
n3	Hepatitis_B
n4	Hepatitis_C
n5	Hepatitis_D
n6	Hepatitis_E
n7	Hbv_acute
n8	Hbv_chronic
n9	Hbv_severe
n10	Hbv_cholestatic
n11	Hbv_cirrhosis

**Table 2:** Similarity between nodes in hepatitis ontology

	n1	n2	n3	n4	n5	n6	n7	n8	n9	n10	n11
n1	1	0.4541	0.4541	0.4541	0.4541	0.4541	0.3145	0.3145	0.3145	0.3145	0.3145
n2	0.4541	1	0.2312	0.2312	0.2312	0.2312	0.2099	0.2099	0.2099	0.2099	0.2099
n3	0.4541	0.2312	1	0.2312	0.2312	0.2312	0.5007	0.5007	0.5007	0.5007	0.5007
n4	0.4541	0.2312	0.2312	1	0.2312	0.2312	0.2099	0.2099	0.2099	0.2099	0.2099
n5	0.4541	0.2312	0.2312	0.2312	1	0.2312	0.2099	0.2099	0.2099	0.2099	0.2099
n6	0.4541	0.2312	0.2312	0.2312	0.2312	1	0.2099	0.2099	0.2099	0.2099	0.2099
n7	0.3145	0.2099	0.5007	0.1799	0.1799	0.1799	1	0.3145	0.3145	0.3145	0.3145
n8	0.3145	0.2099	0.5007	0.1799	0.1799	0.1799	0.3145	1	0.3145	0.3145	0.3145
n9	0.3145	0.2099	0.5007	0.1799	0.1799	0.1799	0.3145	0.3145	1	0.3145	0.3145
n10	0.3145	0.2099	0.5007	0.1799	0.1799	0.1799	0.3145	0.3145	0.3145	1	0.3145
n11	0.3145	0.2099	0.5007	0.1799	0.1799	0.1799	0.3145	0.3145	0.3145	0.3145	1

## Results and discussion

Query expansion is the optimization of the original query. In this paper, the purpose of the experiment is to verify whether the precision of the query is improved or not.

There are two traditional evaluation indicators: recall and precision. Recall is the ratio of the number of retrieved relevant documents to that of all the relevant documents. Precision is the ratio of the number of retrieved correct documents to that of relevant documents retrieved.

Because most of search engine users only pay attention to the feedback of the top N result [15], this paper uses precision@N to evaluate the accuracy of the method of query extension. The calculation of precision@N is as follows:

Precision@N = the number of top N returned relevant documents/N.

Precision@N is the query accuracy of the first N documents. In order to measure the order of top K related documents, the research also uses the other indicator AP@K, whose aim is to describe the average accuracy of relevant documents in the top K documents. The following is the formula for AP@K:

$$ AP@K = \frac{1}{r}\sum\limits_{{rank_{j} \le k}} {\frac{j}{{rank_{j} }}} $$

In the formula, r stands for relevant documents in top k retrieval results. j refers to the first j documents in top k results. rankj means the ordinal of the first j document in result documents.

In this way, precision@N and AP@K together can more fully evaluate the accuracy of the top K retrieval results, which match with most retrieval user’s habits, because most users focus on the top K retrieval results in the retrieval process. Considering that most users generally focus on the top 40 retrieval results, we set N = K = 40.

For the sake of testing the accuracy of this query expansion method, we set up a prototype system of hepatitis domain ontology knowledge base, downloaded from approximately 2000 web pages in the field of medical information database, such as PubMed, Springer Link/medicine and CNKI. PubMed, a free search engine, comprises more than 23 million citations for biomedical literature from MEDLINE, life science journals, and online books. Springer Link provides researchers with access to millions of scientific documents from journals, books, series, protocols and reference works. CNKI, found by Tsinghua University, is an abbreviation of China National Knowledge Infrastructure, which contains more than 7000 journals and supports searches in Chinese and English. The development environment of the prototype system included Eclipse 3.4, and the Jena 2.6.4 semantic framework. Jena is a free and open source Java framework for building semantic web applications, and is composed of different APIs to process ontology data.

This paper randomly selected five terms respectively for searching in form of query expansion, the result of expansion word set is shown in Table 3.

**Table 3:** Expansion word set result

The query number	Query keyword	Expansion word set	Expansion type
1	Viral hepatitis	Viral hepatitis, hepatitis A, hepatitis B, hepatitis C, hepatitis D, hepatitis E	Hyponym
2	Serum hepatitis	Serum hepatitis, hepatitis b	Synonym
3	cirrhosis hepatitis B	Cirrhosis hepatitis B, hepatitis B, serum hepatitis	Hypernym, synonym
4	Acute hepatitis	Acute hepatitis, acute hepatitis A, acute hepatitis B, acute hepatitis C, acute hepatitis D, acute hepatitis E, acute icteric hepatitis and acute non-icteric hepatitis	Hyponym, instance
5	Acute hepatitis B	Acute hepatitis B, acute icteric hepatitis B, acute non-icteric hepatitis B	Instance

**Fig. 10:**
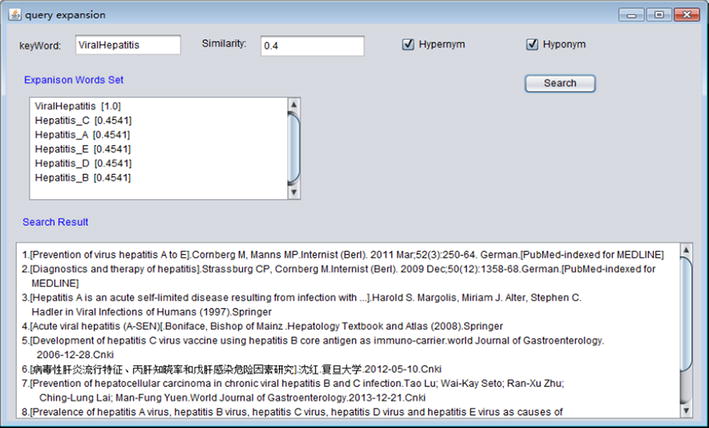
Query expansion interface of hepatitis ontology

Viral hepatitis’ hyponym includes hepatitis a, hepatitis b, hepatitis c, hepatitis d and hepatitis e. By hyponym query expansion, the expansion word set of query No. 1 is viral hepatitis, hepatitis A, hepatitis B, hepatitis C, hepatitis D and hepatitis E. Serum hepatitis is a synonym of is hepatitis B. Through synonym expansion, word set of query No. 2 becomes serum hepatitis and hepatitis B. Cirrhosis hepatitis B is the subclass of hepatitis. According to similarity computing, the calculation result of similarity between the word “cirrhosis hepatitis” and “hepatitis B” is greater than others (0.5007). The expansion word set of query No.3 is Cirrhosis hepatitis B, hepatitis B, serum hepatitis. In the hepatitis ontology, acute hepatitis includes five subclasses and two instances. After expansion, the word set of query No. 4 is acute hepatitis, acute hepatitis A, acute hepatitis B, acute hepatitis C, acute hepatitis D, acute hepatitis E, acute icteric hepatitis and acute non-icteric hepatitis. Acute hepatitis B has two instances in ontology hierarchy, so the expansion word set of query No. 5 is acute hepatitis B, acute icteric hepatitis B, acute non-icteric hepatitis B. Figure 10 is the software interface of query expansion. Users input a keyword, similarity and expansion type, system can automatically output expansion word set and search results.

**Fig. 11:**
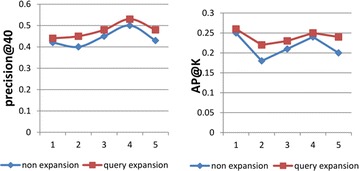
Comparison of query expansion results

Figure 11 shows that the accuracy of query expansion is improved in both precision@40 and AP@40, which indicates that query expansion improves the accuracy of the query after using the method proposed in this paper. No matter which indicator used between precision@40 and AP@40, the increase percentage of query No. 2 is clear. In Table 4, the percentage increase for query No. 2 is respectively 21 and 11 %, and this indicates the positive effect of synonym expansion query. In addition, the percentage increase of query No. 5 ranks in second place. The main reason is that instance of class in hepatitis ontology is more helpful to narrow the searching scope after instance query expansion.

**Table 4:** Query expansion result evaluation

Metric	Method	1	2	3	4	5
Precision@40	Non expansion	0.42	0.4	0.45	0.5	0.43
	Query expansion	0.44 (↑5 %)	0.45 (↑13 %)	0.48 (↑7 %)	0.53 (↑6 %)	0.48 (↑12 %)
AP@40	Non expansion	0.25	0.18	0.21	0.24	0.2
	Query expansion	0.26 (↑4 %)	0.22 (↑22 %)	0.23 (↑10 %)	0.25 (↑4 %)	0.24 (↑20 %)

## Conclusions

In this paper we have presented the hepatitis ontology construction process. We have also presented a semantic query expansion method for hepatitis ontology, which applies synonym expansion, hypernym/hyponym expansion, and similar word expansion. Additionally, we have adopted semantic similarity computing to improve retrieval performance. Experiments show that search precision of query expansion is higher based on domain concept relationship.

Experimental results show that different types of query expansion are of varying effectiveness on the effects of query expansion. Future work will focus on the contribution of different types of query expansion to the query results. Another research direction will be to find the relationships among the liver disease ontology classification hierarchy, concept granularity, and accuracy of query expansion.
